# Exercise Promotes Hippocampal Neurogenesis in T2DM Mice via Irisin/TLR4/MyD88/NF-κB-Mediated Neuroinflammation Pathway

**DOI:** 10.3390/biology13100809

**Published:** 2024-10-10

**Authors:** Haocheng Xu, Xin Tian, Yuanxin Wang, Junjie Lin, Baishu Zhu, Chen Zhao, Bin Wang, Xin Zhang, Yu Sun, Nan Li, Xun Sun, Fanxi Zeng, Mingzhi Li, Xiquan Ya, Renqing Zhao

**Affiliations:** College of Physical Education, Yangzhou University, Yangzhou 225127, China; haochengxu2023@163.com (H.X.); tianxin96331@163.com (X.T.); wangyuanxin880330@163.com (Y.W.); junjielin2022@163.com (J.L.); baishuzhu@outlook.com (B.Z.); zc072866@outlook.com (C.Z.); bwang1999@126.com (B.W.); xxinz1998@163.com (X.Z.); 15050599442@163.com (Y.S.); ln991120@outlook.com (N.L.); mz120220792@stu.yzu.edu.cn (X.S.); 15048380478@163.com (F.Z.); lifanlilefan@163.com (M.L.); y13753915464@163.com (X.Y.)

**Keywords:** diabetes, cognition, adult hippocampal neurogenesis, exercise, neuroinflammation

## Abstract

**Simple Summary:**

We explored the role of exercise in promoting hippocampal neurogenesis and memory function in T2DM mice through the Irisin/TLR4/MyD88/NF-κB signaling pathway. Ten weeks of exercise training significantly reduced inflammation in the DG region caused by T2DM and promoted hippocampal neurogenesis and memory function. However, these positive effects could be reversed by treatment with Cyclo RGDyk, which blocks irisin receptor signaling. The results suggest that exercise may be an effective strategy for alleviating cognitive decline caused by diabetes by activating the irisin signaling pathway.

**Abstract:**

Neuroinflammation is a major feature of type 2 diabetic mellitus (T2DM), adversely affecting hippocampal neurogenesis. However, the precise mechanism is not fully understood, and therapeutic approaches are currently lacking. Therefore, we determined the effects of exercise on neuroinflammation and hippocampal neurogenesis in T2DM mice, with a specific focus on understanding the role of the irisin and related cascade pathways in modulating the beneficial effects of exercise in these processes. Ten-week exercise significantly decreased T2DM-induced inflammation levels and markedly promoted hippocampal neurogenesis and memory function. However, these positive effects were reversed by 10 weeks of treatment with cyclo RGDyk, an inhibitor of irisin receptor signaling. Additionally, exercise helped reduce the M1 phenotype polarization of hippocampal microglia in diabetic mice; this effect could be reversed with cyclo RGDyk treatment. Moreover, exercise markedly increased the levels of fibronectin type III domain-containing protein 5 (FNDC5)/irisin protein while decreasing the expression of Toll-like receptor 4 (TLR4), myeloid differential protein-88 (MyD88), and nuclear factor kappa-B (NF-κB) in the hippocampus of T2DM mice. However, blocking irisin receptor signaling counteracted the down-regulation of TLR4/MyD88/NF-κB in diabetic mice undergoing exercise intervention. Conclusively, exercise appears to be effective in reducing neuroinflammation and enhancing hippocampal neurogenesis and memory in diabetes mice. The positive effects are involved in the participation of the irisin/TLR4/MyD88/NF-κB signaling pathway, highlighting the potential of exercise in the management of diabetic-induced cognitive decline.

## 1. Introduction

T2DM, characterized by insulin resistance, is predicted to affect over 783 million individuals by 2045 [1]. This systemic disease can lead to cognitive decline and is associated with a higher risk of dementia [2,3]. One of the essential factors contributing to memory degradation and neurological diseases is the impairment of the neurogenic niche in the hippocampus, which leads to reduced adult hippocampal neurogenesis (AHN) [4,5,6]. Diabetes can affect various aspects of AHN, including the number of neural stem cells, the production of new neurons, and the process of maturation and integration of neurons into the existing network [7]. Therefore, understanding how diabetes affects AHN and cognition, and developing approaches to target the diabetic brain, is essential for managing cognitive impairments in diabetes.

Chronic inflammation in the brain is a hallmark of diabetes [8]. Microglia, the brain’s immune sentinels, are crucial in initiating the inflammatory response to infection or tissue damage [9]. When microglia encounter harmful stimuli, they respond by producing inflammatory cytokines such as tumor necrosis factor-α (TNF-α) and interleukin-1β (IL-1β) [10]. These molecules are essential for polarizing microglia into the classically activated “M1” state—a protective response that typically subsides once the infection is resolved [11]. However, uncontrolled, long-term inflammation can lead to tissue damage [12], subsequently affecting AHN [13]. In contrast, M2-polarized microglia contribute to improving the brain microenvironment by producing anti-inflammatory cytokines like transforming growth factor-β (TGF-β) [14]. This anti-inflammatory response has the potential to manage AHN and cognitive dysfunction associated with diabetes.

Currently, exercise is widely recognized as a crucial non-pharmacological approach for preventing or managing chronic conditions such as diabetes and neurodegenerative disorders [15,16,17]. For example, exercise can improve insulin resistance, lower hyperglycemia, and enhance metabolism, thereby alleviating diabetes-related symptoms [16]. Additionally, exercise has a profound positive impact on the brain, particularly in promoting neurogenesis [18,19]. It can stimulate the generation of newborn neurons, increase neuronal progenitor cells, and facilitate the maturation and integration processes in the mouse hippocampus, improving the brain’s plasticity and cognitive functions [20]. Accordingly, exercise can ameliorate cognitive deficits such as Alzheimer’s disease (AD) by promoting neurogenic factors, reducing neuroinflammation, and supporting neuronal survival, maturation, and synaptic plasticity [21,22]. Moreover, exercise notably suppresses the inflammatory response following STZ injury and effectively transforms activated microglia from the pro-inflammatory M1 phenotype to the anti-inflammatory M2 phenotype [23]. These findings suggest that exercise could serve as an effective strategy for improving diabetes and mitigating associated complications including cognitive impairment partly by reducing inflammation [24]. However, the mechanisms underlying its neuroprotective effects remain incompletely understood, highlighting a gap in our current knowledge within this research field.

Exercise affects neuroinflammation and AHN through various molecular pathways, such as irisin and the TLR4/MyD88/NF-kB. Irisin, identified as a new myokine, is closely linked to exercise and is generated through the proteolytic cleavage of the N-terminal segment of FNDC5 [25]. Irisin administration effectively hinders the M1 phenotype polarization of microglia while promoting the M2 phenotype polarization [26]. Accordingly, irisin can serve a neuroprotective function in brain disorders like AD and improve cognitive performance [27]. Irisin is regarded to function through binding its putative receptor, αVβ5, a process that can be inhibited by cyclo RGDyk, thereby blocking the signal transduction and gene expression triggered by irisin [28]. Wei and colleagues have demonstrated that c(RGDyK)-modified liposomes can enhance their permeability through the BBB by binding to integrin receptors, as documented in their study [29]. The structural integrity of the BBB is progressively compromised in T2DM, as supported by multiple research findings [30,31]. Given the lipophilic nature and the relatively small molecular weight of 847.72 Daltons of Cyclo RGDyK, we hypothesize that this compound may be capable of penetrating the compromised BBB in diabetic conditions, potentially serving to block the effects of irisin.

Another key molecule is TLR4, where irisin plays a significant role upstream of TLR4 [32,33]. TLR4 is not only involved in the activation of the NLRP3 inflammasome [34] but also has a crucial impact on neuroinflammation and neurogenesis through the MyD88/NF-κB axis [35,36]. Under the stimulation of environmental factors such as hyperglycemia, the TLR4 receptor on the surface of microglia is activated [37], thereby triggering the classic MyD88/NF-κB signaling pathway [38]. The activation of this pathway leads to the release of pro-inflammatory cytokines such as TNF-α and IL-1β [39]. These cytokines then enhance the inflammatory response through their respective receptors, such as the IL-1 receptor or TNF receptor, creating a vicious cycle that exacerbates the inflammatory process [40,41].

Interestingly, genetic down-regulation of TLR4 expression shows protective effects in diabetic mice [42]. Current evidence points to irisin as a key factor in facilitating the link between physical exercise and the enhancement of brain flexibility and memory [43]. Furthermore, the positive impacts of exercise on reducing neuroinflammation are partially achieved by inhibiting the TLR signaling pathway [44]. However, the interplay between exercise, irisin, and TLR4 signaling in modulating neuroinflammation and neurogenesis in T2DM remains unclear. Therefore, this study aimed to determine the effects of exercise on neuroinflammation and hippocampal neurogenesis in T2DM mice, with a specific focus on understanding the role of the irisin and TLR4/MyD88/NF-κB pathway in modulating the beneficial effects of exercise in these processes.

## 2. Methods

### 2.1. Animal Experimentation

**Figure 1:**
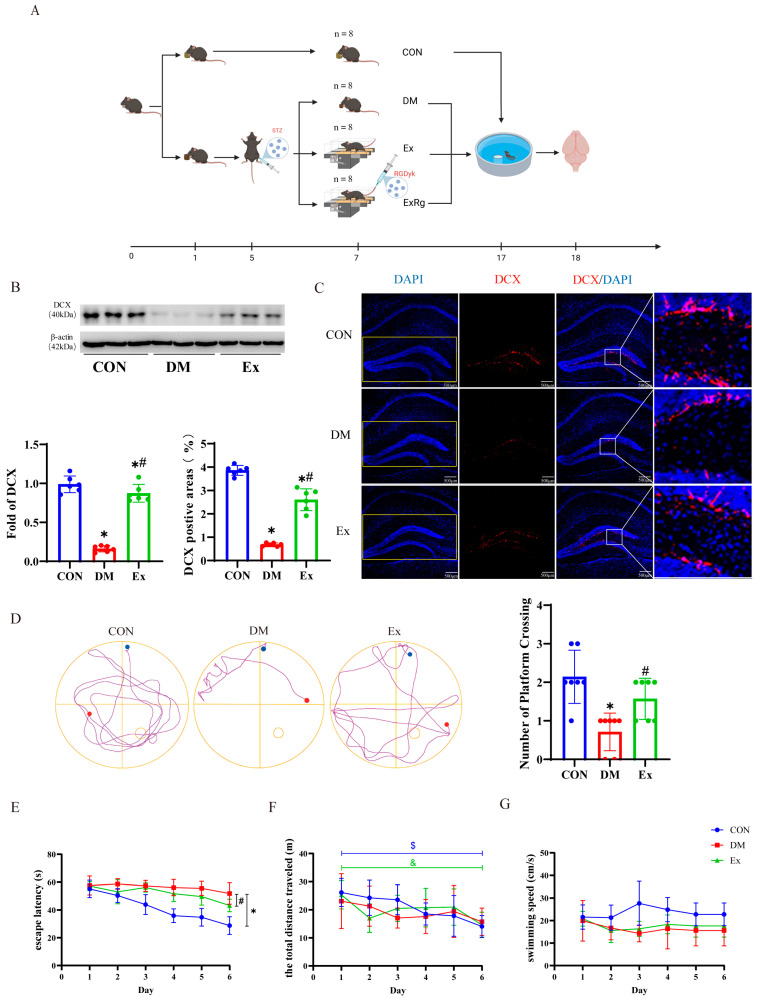
Timeline of the experimental procedures and the impact of exercise on hippocampal neurogenesis in diabetic mice. (**A**) The schematic depicts the timeline of the experimental procedures and the organization of the experimental groups. The mice were injected with streptozotocin (STZ) or sodium citrate buffer at 8 weeks of age to induce T2DM. After the induction of T2DM, the mice were allocated to the following groups: control (n = 8), diabetes (n = 8), diabetes with exercise (n = 8), and diabetes with exercise plus cyclo RGDyk treatment (n = 8). The regimen of treadmill exercise and cyclo RGDyk administration was maintained over 10 weeks. At the end of the experiment, all groups underwent the Morris water maze test and were then used for immunofluorescence and other analyses. (**B**) Western blot imagery and quantitative analysis of Iba1 expression in the hippocampus (n = 6). (**C**) Representative confocal images of DCX (red) and DAPI (blue) staining in the hippocampus, accompanied by the area of positive area expression of DCX (%) (n = 6). Scale bar = 500 µm. (**D**) The swimming trajectories and the number of platform crossings in the Morris water maze test (n = 8). (**E**) Escape latency (n = 8). (**F**) The total distance traveled (n = 8). (**G**) Swimming speed (n = 8). Data are represented as ± SD; * denotes significance (*p* < 0.05) compared to the CON group; # denotes significance (*p* < 0.05) compared to the DM group; $ and & denote significance (*p* < 0.05) between day 6 and day 1.

The reporting of animal experiments follows the recommendations in the ARRIVE guidelines. The temporal progression of the experiment is delineated as depicted in Figure 1A. Forty male C57BL/6 mice (8 weeks old), procured from the Experimental Animal Center of Yangzhou University, were maintained under specific pathogen-free conditions at a temperature of 24 ± 2 °C and relative humidity of 60 ± 10%, with a 12 h light/dark cycle. All animal procedures were conducted in accordance with the approved animal protection and use guidelines of Yangzhou University (Approval No. 202303133). After the successful creation of the T2DM model, diabetes together with control mice were allocated randomly to the following groups: (1) control (Con, N = 8); (2) diabetes mellitus (DM, N = 8); (3) diabetes plus exercise (Ex, N = 8); and (4) diabetes plus exercise and cyclo RGDyk (an antagonist of the irisin receptor -αVβ5) (ExRg, N = 8). Body weight and fasting blood glucose (FBG) levels were measured weekly. After the last exercise training, all mice were anesthetized and euthanized with Zoletil 50 (20 mg/kg) in each group in order to collect samples.

### 2.2. T2DM Models

The protocol for establishing T2DM mouse models has been described previously [45]. Briefly, to establish the T2DM model in male C57BL/6 mice, a high-fat diet (HFD) was gradually introduced, comprising 68% regular feed, 20% sucrose, 10% lard, 1.5% cholesterol, and 0.5% bile salts. The control group was maintained on a standard diet. After 4 weeks of the HFD, a single dose of STZ (Sigma, MO, USA) (100 mg/kg) dissolved in citrate buffer (pH 4.4) was administered intraperitoneally following a 12 h fast. The control group received an equivalent dose of sodium citrate buffer. Successful modeling was confirmed by FBG levels ≥ 16.7 mmol/L [46]. We conducted a T2DM model induction in 32 C57BL/6 mice, with a successful modeling rate of 75%, thus resulting in 24 mice successfully modeled.47

### 2.3. Drug Administration Protocol

Two weeks after establishing the diabetes model, mice in the ExRg group were intravenously injected with cyclo RGDyk (Glpbio, CA, USA) at a dose of 2.5 mg/kg, twice weekly for 10 weeks. The other groups received equivalent doses of a 10% dimethyl sulfoxide solution [28].

### 2.4. Exercise Training Protocol

For aerobic treadmill training, mice underwent 10 weeks of treadmill training at a 5% incline, 5 days per week. The training duration gradually increased from 10 to 60 min during the first week of acclimatization, with the speed gradually increasing from 8 to 12 m/min. From the second to the tenth week, a fixed 50 min treadmill training regimen was followed at a speed of 12 m/min, including a 5 min warm-up and a 5 min cool-down.

### 2.5. Morris Water Maze (MWM) Test

The MWM test is conducted in a distinct blue circular pool, segregated into four quadrants. This unique design allows for a comprehensive evaluation of the subjects’ cognitive abilities. Over a span of six consecutive days, four trials were performed daily, each lasting 60 s, providing the mice an opportunity to locate a hidden platform. A 10 s rest period on the platform ensured equal observational time for spatial cues post-trial for each mouse. Subsequently, a probe trial was conducted to assess spatial memory retention. The platform was removed, and the mice were allowed to swim for 60 s from the same starting point. The number of times the mice crossed the location where the platform was previously situated was carefully recorded. The MWM test was conducted using a water maze device produced by Zhenghua Biological Instrument Co., Ltd. (Chengdu, China) and the relevant data were collected and analyzed using the ANY-maze software (version 7.15).

### 2.6. Immunofluorescence Staining

After 10 weeks of exercise, the mice were injected with cold PBS through cardiac puncture, and then the brains were removed and divided into two halves along the midline. Each group consisted of six left hemispheres used for immunofluorescence staining. They were sequentially immersed in 4% paraformaldehyde, 20% sucrose, and 30% sucrose for fixation and dehydration. Coronal sections (30 μm thick) were cut using a cryostat (CM1950, Leica, Wetzlar, Germany) and placed on adhesive slides. After washing in PBS, the sections were incubated in PBS + 1% Triton to permeabilize the cell membranes and blocked with 10% goat serum for 1 h. The sections were then incubated overnight at 4 °C with the following primary antibodies: Rabbit anti-Iba-1 (1:100, ET1705-78, HuaBio, Hangzhou, China); Mouse anti-DCX (1:100, sc-271390, Santa Cruz, CA, USA); Mouse anti-CD206 (1:100, sc-376108, Santa Cruz, USA); and Mouse NOS2 (1:100, SC-7271, Santa Cruz, USA). After washing three times in PBS, the sections were incubated for 2 h at room temperature with secondary antibodies conjugated with Alexa Fluor-488/594/647 (1:200 Biodragon, Suzhou, China). Nuclei were stained with 4′,6-diamidino-2-phenylindole (DAPI). The sections were then observed and imaged under a super-resolution laser confocal microscope (TSC SP8 STED, Leica). For the quantification of immunofluorescence, we first selected the DG region of the hippocampus within the yellow square frame using ImageJ. For images with a single stain, we calculated the positive area percentage by dividing the area of protein positivity by the area of DAPI. For images with dual staining, we determined the positive area percentage by dividing the area where the two fluorescence signals intersect by the area where the two fluorescence signals combine.

### 2.7. Western Blot Analysis

After 10 weeks of exercise, the mice were perfused with cold PBS under deep anesthesia. The brain samples were quickly extracted, and the brain tissues were divided into two halves along the midline. Each group consisted of six right hemispheres that were rapidly frozen in liquid nitrogen and stored at −80 °C. For Western blot detection, the hippocampal tissue samples were taken out from the brains, homogenized in RIPA lysis buffer (G2002, Servicebio, Wuhan, China), and centrifuged at 4 °C at 15,000× *g* for 20 min. Equal amounts of protein were loaded onto SDS-PAGE gels and run by electrophoresis, then transferred to nitrocellulose membranes. The membranes were blocked and then incubated overnight at 4 °C with the following primary antibodies: Rabbit anti-FNDC5 (1:1000, A18107, Abclonal, Wuhan, China); Rabbit anti-TLR4 (1:500, WL00196, wanleibio, LN, China); Rabbit anti-MyD88 (1:500, WL02494, wanleibio, China); Mouse anti-NFκB (1:1000, sc-8008, Santa Cruz, CA, USA); Mouse anti-p-NF-κB (1:1000, sc-136548, Santa Cruz, USA); Rabbit anti-IL-1β (1:200, WL00891, wanleibio, China); Rabbit anti-Iba-1 (1:1000, AF7143, Beyotime, Shanghai, China); Mouse anti-DCX (1:500, sc-271390, Santa Cruz, USA); and Rabbit anti-TNF-α (1:1000, WL01581, wanleibio, Shanghai, China). The membranes were incubated with mouse anti-β-actin (1:2000, GB15001, Servicebio, China) as a loading control. Appropriate secondary antibodies (1:4000, Biodragon) were incubated with the membranes for 1 h at room temperature. Bands were detected using the NcmECL High chemiluminescent reagent kit (Ncmbio, Suzhou, China) and observed with an imaging system (CLINX, ChemiSciope 6200, Shanghai, China). The relative density of protein immunoblot images was analyzed using ImageJ software (NIH, USA, version 1.54).

### 2.8. Quantitative RT-PCR

The mRNA expression levels of IL-1β and TNF-α were detected using qRT-PCR. Briefly, the total RNA was isolated from the hippocampal tissues of the mice in each group using the TRIzol reagent (vazyme, Nanjing, China). The total RNA was then reverse transcribed into cDNA using the SuperScript III reverse transcription kit (vazyme, China). qRT-PCR was conducted using the SYBR Green qPCR SuperMix (vazyme, China) on a real-time fluorescence quantitative PCR system (7500 Fast, Thermo, Waltham, MA, USA). The primers used in this study are listed in Table 1. The results were analyzed using the 2^−ΔΔCt^ method.

### 2.9. Statistical Analysis

The data are articulated as the mean ± standard deviation (SD). Prior to analysis, the normality of the data distribution and homogeneity of variance were evaluated. For comparisons between two groups, if these assumptions were satisfied, a one-way analysis of variance (ANOVA) was employed to assess intergroup disparities. In instances where these assumptions were violated, suitable non-parametric tests were utilized. All statistical analyses were conducted using GraphPad Prism software (version 9.4.1). The data were garnered from a minimum of three independent experiments, each conducted under identical conditions. A *p*-value less than 0.05 was deemed statistically significant.

## 3. Results

### 3.1. Hippocampal Neurogenesis Is Impaired in Diabetes but Restored after Exercise Intervention

T2DM is a common chronic condition that impacts billions of individuals and can lead to various symptoms, such as heart disease, hypertension, kidney disease, cancer, and even death [47]. Recent evidence suggests that long-standing diabetes may also contribute to mild cognitive impairment (MCI) and raise the risk of AD [48,49]. Despite this, there are currently no effective methods for preventing or managing MCI in people with diabetes. However, mounting evidence suggests that exercise not only helps to improve metabolic dysfunctions, insulin resistance, and hyperglycemia [50], but also increases blood flow to the brain, reduces neuroinflammation, and promotes memory function [51]. In this study, we tested whether the improvement of memory function is a result of elevated hippocampal neurogenesis. We employed Western blot techniques and immunofluorescence staining to measure the alterations in AHN in T2DM mice and the potential influence of a 10-week physical exercise regimen. The findings indicated that the hippocampal protein expression of DCX in the diabetes mice was markedly lower than those in the control group (Figure 1B). However, the decreased DCX protein levels were significantly restored after 10 weeks of treadmill exercise (Figure 1B and Figure S1). Moreover, it is of particular interest that compared with the CON group, DCX-positive areas in the DG of the hippocampus were significantly decreased in the DM group (Figure 1C). However, the Ex group had more DCX-positive area in the hippocampus than the DM group (Figure 1C). In the Morris water maze test, we observed a significant reduction in the number of platform crossings in the diabetic group compared to the control group, implying that diabetes may adversely affect the learning and memory capabilities of the mice. However, following the tenth week of treadmill exercise training, the number of platform crossings by the diabetic mice noticeably increased (Figure 1D), suggesting that regular exercise may contribute to the amelioration of cognitive impairments caused by diabetes. During the training trial period, the results indicated that mice in the DM group exhibited an increase in escape latencies compared to those of the corresponding CON group (Figure 1E), and escape latencies increases were reversed by 10 weeks of aerobic exercise (Figure 1E). Moreover, after six days of training. The total distance traveled by mice in both the CON and Ex groups significantly decreased compared to the first day, while there was no significant change in the swimming distance of mice in the DM group (Figure 1F). Additionally, there was no significant difference in swimming speed between the three groups on the first and sixth days (Figure 1G), indicating that the diabetic mice did not experience impairment in their motor function. This suggests that learning and memory functions were impaired by STZ induction, and 10 weeks of aerobic exercise can mitigate these symptoms. These findings underscore the crucial role of exercise training in promoting hippocampal neurogenesis and enhancing cognitive function in individuals with diabetes.

### 3.2. Exercise Alleviates Neuroinflammation in the Hippocampus

**Figure 2:**
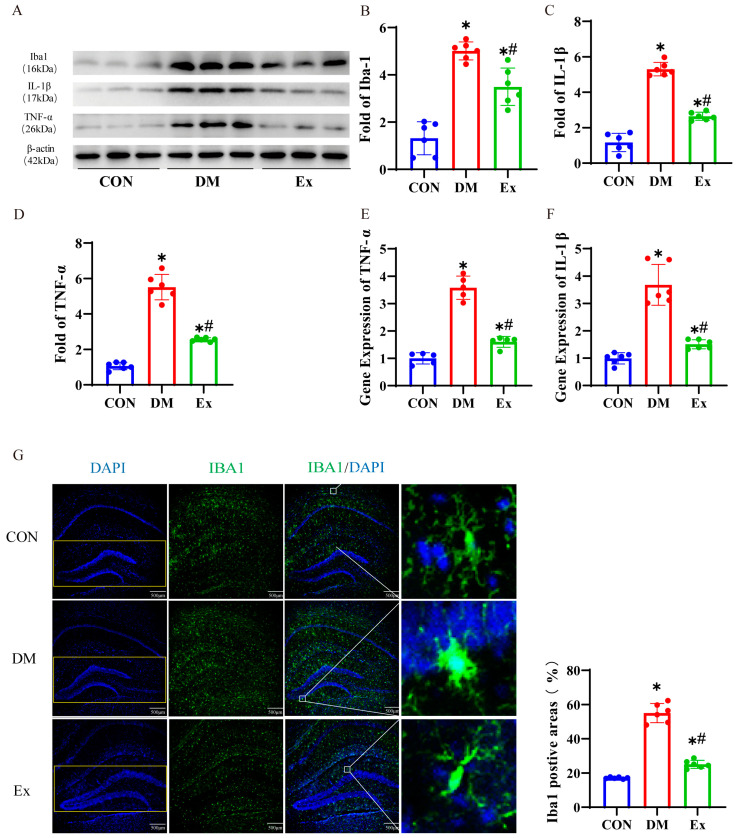
The impact of exercise on hippocampal neuroinflammation in diabetes. (**A**) Representative Western blot images of Iba1, IL-1β, and TNF-α expression in the hippocampus. (**B**–**D**) Quantification of Iba1, IL-1β, and TNF-α expression in the hippocampus of the mice. (**E**,**F**) Relative mRNA levels of TNF-α and IL-1β in the hippocampus of the mice. (**G**) Representative confocal images of Iba1 (green) and DAPI (blue) staining in the hippocampus, accompanied by the area of positive area expression of Iba1 (%). Scale bar = 500 µm. n = 6 mice per group. Data are represented as ± SD. * denotes significance (*p* < 0.05) compared to the Con group. # denotes significance (*p* < 0.05) compared to the DM group.

Previous studies suggested that a persistent hyperglycemic condition in diabetic mellitus is a pivotal trigger for hippocampal neuroinflammation [52], primarily via the excessive activation of microglial cells, which leads to the release of inflammatory substances [10]. Neuroinflammation frequently results in deleterious alterations of the neurogenic niche that may lead to the impairment of AHN and cognitive dysfunction [53]. However, it is still unclear whether exercise could ameliorate neuroinflammation and reduce the production of pro-inflammatory cytokines in the hippocampal of diabetic mice. Our results showed that the Iba1 positive area in the DG subregion of the hippocampus was significantly increased in the DM group compared with the CON group (Figure 2G). However, the Ex group had fewer Iba1-positive areas in the hippocampus than the DM group (Figure 2G). We found that microglia in the CON group exhibited a more complex branched morphology, while those in the DM group displayed fewer branches and a morphology closer to the amoeboid shape. Importantly, exercise promoted the transformation of microglia towards a more complex branched morphology (Figure 2G). In addition, the protein levels of Iba-1 in the hippocampus of T2DM mice were significantly elevated compared to the control group, whereas exercise training significantly reduced the levels of Iba-1 (Figure 2A,B and Figure S2). Similarly, we observed a marked increase in the levels of TNF-α and IL-1β proteins in the hippocampus of T2DM mice compared to the control group (Figure 2A,C,D and Figure S2), indicating the presence of pronounced hippocampal neuroinflammation in diabetic mice. To further probe the level of inflammation within the hippocampal region, quantitative PCR was conducted to evaluate the mRNA expression levels of inflammatory markers TNF-α and IL-1β. Compared to the control group, the diabetic group demonstrated a significant elevation in the mRNA expression levels of TNF-α and IL-1β (Figure 2E,F). However, 10 weeks of treadmill running significantly ameliorated hippocampal inflammation levels (Figure 2E,F). This indicates that aerobic exercise could potentially reverse the inflammatory conditions in the hippocampal neurogenic niche in diabetic mice.

### 3.3. Exercise Affects Microglial Activation Patterns in the Hippocampus

**Figure 3:**
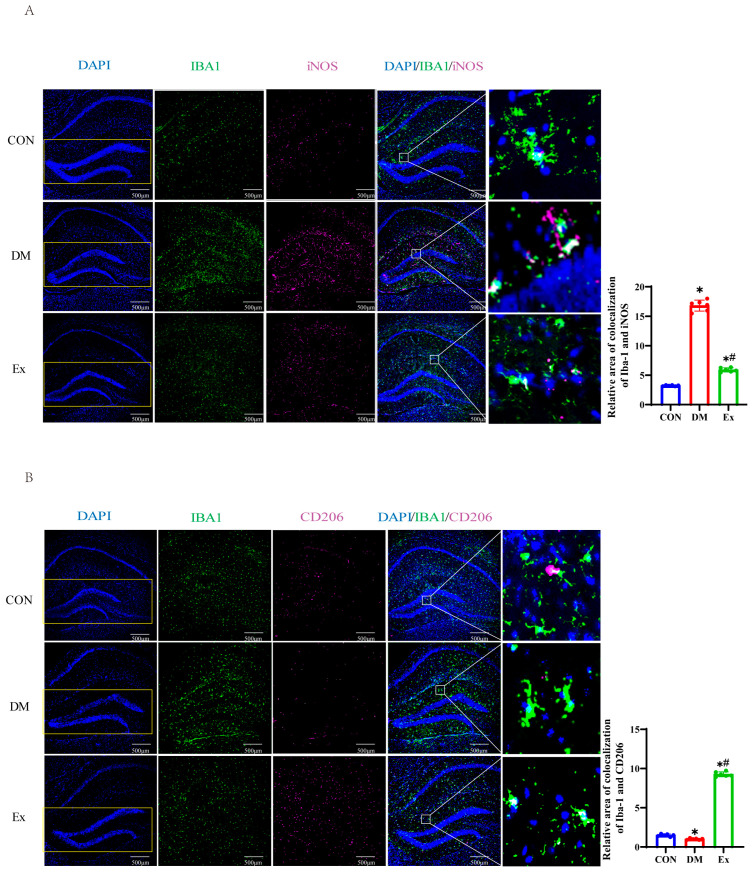
The beneficial impact of exercise-induced irisin on the polarization of microglial cells in the diabetic hippocampus. (**A**) Representative confocal images of Iba1 (green), iNOS (magenta), and DAPI (blue) staining in the hippocampus, along with the count of co-localized positive cells. Scale bar = 500 µm. (**B**) Representative confocal images of Iba1 (green), CD206 (magenta), and DAPI (blue) staining in the hippocampus, along with the count of co-localized positive cells. Scale bar = 500 µm. n = 6 mice per group. Data are represented as ± SD. * denotes significance (*p* < 0.05) compared to the CON group, # denotes significance (*p* < 0.05) compared to the DM group.

Microglial cells are the brain’s innate immune cells that help maintain the central nervous system’s balance by detecting and eliminating pathogens and exhibiting anti-inflammatory effects [54]. However, prolonged exposure to high blood sugar levels and metabolic dysfunction can lead to the accumulation of pathological microglial cells and the excessive production of pro-inflammatory cytokines [13,55]. Reversing the pro-inflammatory phenotype (M1 microglia) to an anti-inflammatory pattern (M2 microglia) is suggested as an effective way to reduce neuroinflammation. Therefore, we investigated whether exercise could shift the microglial pattern from M1 to M2 in diabetic mice. Our findings indicated that, in comparison to the control group, the Iba1^+^iNOS^+^ positive area in diabetic mice was significantly increased, whereas exercise intervention markedly decreased these cell numbers (Figure 3A). Conversely, we observed that the Iba1^+^CD206^+^ positive area in the exercise group of diabetic mice was significantly higher than in the diabetic group (Figure 3B). These results suggest that exercise may protect the hippocampal microenvironment in diabetic mice by promoting the transition of microglial cells from an M1 pro-inflammatory phenotype to an M2 anti-inflammatory phenotype.

### 3.4. Exercise Decreases TLR4/MyD88/NF-kB Pathways but Is Inhibited by Cyclo RGDyk Treatment

**Figure 4:**
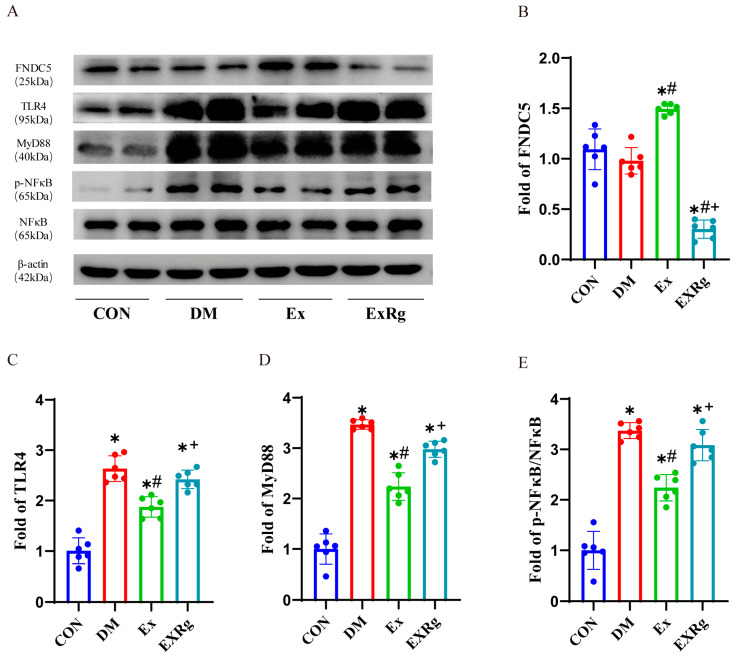
Injection of cyclo RGDyk reverses the beneficial effects of exercise-induced irisin on the hippocampus in diabetes. (**A**) Representative Western blot images of FNDC5, TLR4, MyD88, and p-NF-κB/NF-κB expression in the hippocampus. (**B**–**E**) Quantification of FNDC5, TLR4, MyD88, and p-NF-κB/NF-κB expression in the hippocampus of the mice. Data are represented as ± SD. n = 6 mice per group. * indicate significance (*p* < 0.05) compared to the CON group, # denote significance (*p* < 0.05) compared to the DM group, and + denote signify significance (*p* < 0.05) compared to the Ex group.

Previous research has indicated that the activation of microglial cells and the increased production of pro-inflammatory cytokines are partially controlled by TLR pathways [56]. Therefore, we investigated whether exercise could impact TLR signaling pathways and subsequently alleviate neuroinflammation. Our results indicated that the protein levels of TLR4, MyD88, and p-NF-κB/NF-κB in the hippocampus of diabetic mice were significantly higher compared to the control (Figure 4A–E and Figure S3). However, these elevated protein levels were notably reduced after 10 weeks of treadmill running, indicating that exercise may suppress TLR4/MyD88/NF-κB inflammatory pathways (Figure 4A–E and Figure S3). Additionally, exercise training led to a significant increase in irisin levels. Recent studies have suggested that irisin, a novel myokine, plays a role in regulating the relationship between exercise and brain health by reducing neuroinflammation [57]. To verify the role of irisin in regulating the beneficial effects of exercise on microglial activation and cytokine production, we blocked irisin pathways using cyclo RGDyk in exercising diabetic mice. Blocking the irisin signaling pathways increased the reduced levels of TLR4, MyD88, and p-NF-κB/NF-κB in diabetic mice undergoing exercise training A–E and Figure S3). This indicates that irisin receptor signaling might be involved in the protective effects of exercise on TLR pathways.

### 3.5. Cyclo RGDyk Treatment Reverses Microglial Activation and Hippocampal Neurogenesis Impairment

**Figure 5:**
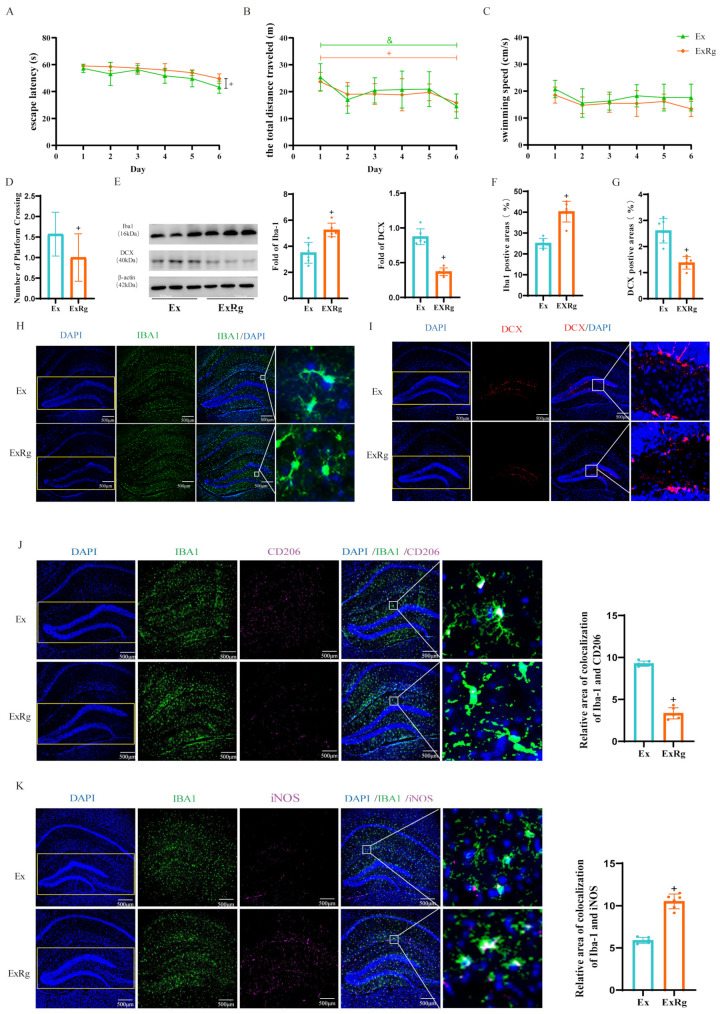
The injection of cyclo RGDyk counteracts the beneficial effects of exercise-induced irisin on microglial cells and adult neurogenesis in the hippocampus of diabetic mice. (**A**) Escape latency (n = 8). (**B**) The total swimming distance from four water maze tests each day for the first six days. (n = 8). (**C**) Swimming speed (n = 8). (**D**) The number of platform crossings in the Morris water maze test (n = 8). (**E**) Representative Western blot images of Iba1 and DCX expression in the hippocampus (n = 6). (**F**,**H**) Representative confocal images of Iba1 (green) and DAPI (blue) staining in the hippocampus, accompanied by the area of positive area expression of Iba1 (%) (n = 6). Scale bar = 500 µm. (**G**,**I**) Representative confocal images of DCX (red) and DAPI (blue) staining in the hippocampus, accompanied by the area of positive area expression of DCX (%) (n = 6). Scale bar = 500 µm. (**D**,**E**) Quantification of Iba1 and DCX expression in the hippocampus of the mice (n = 6). (**J**) Representative confocal images of Iba1 (green), CD206 (magenta), and DAPI (blue) staining in the hippocampus, along with the count of co-localized positive cells (n = 6). (**K**) Representative confocal images of Iba1 (green), iNOS (magenta), and DAPI (blue) staining in the hippocampus, along with the count of co-localized positive cells (n = 6). Data are represented as ± SD. + denote significance (*p* < 0.05) compared to the Ex group.

TLR4/MyD88/NF-κB signaling is a key pathway that regulates the activation of microglia and the production of pro-inflammatory cytokines. We further determined whether blocking the irisin receptor signaling could affect the beneficial effects of exercise on microglial activation and hippocampal neurogenesis. The results showed significant impairments in learning and memory functions in the ExRg group compared to the Ex group (Figure 5A–D). Furthermore, the ExRg group exhibited a significant increase in the Iba1-positive area and a decrease in the DCX-positive area within the DG (Figure 5F–I). Additionally, in comparison to the Ex group, the levels of hippocampal Iba-1 protein expression in the mice receiving exercise training and administrating cyclo RGDyk were significantly increased (Figure 5E and Figure S2), while the DCX protein levels were significantly decreased (Figure 5E and Figure S1). Furthermore, immunofluorescence staining results showed that the administration of cyclo RGDyk markedly reduced the Iba1^+^CD206^+^ positive area (Figure 5J) while it significantly increased the Iba1^+^iNOS^+^ positive area (Figure 5H). These results indicated that the learning and memory deficits observed in the ExRg group may be due to the inhibition of irisin signaling, which affects the exercise-induced shift from M1 to M2 microglial phenotypes and the subsequent enhancement of hippocampal neurogenesis.

## 4. Discussion

In our study, we determined the possible mechanisms underlying the neuroprotective benefits of exercise in a diabetic mouse model. The results demonstrated that exercise can promote the transformation of hippocampal microglial cells towards the M2 phenotype in diabetic mice, while also enhancing AHN. Moreover, the findings suggest that exercise exerts its beneficial effects through the activation of the irisin signaling pathway, which, in turn, inhibits the TLR4/MyD88/NF-κB pathway, thereby mitigating the exacerbation of hippocampal neuroinflammation and the decreases in AHN induced by diabetes.

Exercise is widely recognized as a non-pharmacological approach to enhancing cognitive function [58]. It promotes neurogenesis in the hippocampal region of both healthy and AD adult mice, thereby improving cognitive performance [20,21,22]. However, the effects of exercise in the context of diabetes, and the underlying mechanisms, remain to be addressed. A study by Rahmati suggested that exercise training serves as an efficient approach to preventing and alleviating neuroinflammation and cognitive dysfunction in T2DM [59]. Our results support this notion, demonstrating that exercise effectively reduces inflammation levels in the hippocampus of diabetic mice and significantly enhances AHN leading to cognitive improvement. It indicates that the beneficial impact of exercise on cognition in diabetes may, in part, be attributed to its role in regulating neuroinflammation and subsequently promoting neurogenesis. Diabetes can compromise the cerebral microenvironment and provoke persistent inflammation, both of which are key contributors to learning and memory deficits [60]. Recent research has indicated that neuroinflammation negatively affects neurogenesis, resulting in cognitive decline [24,61]. This suggests that managing neuroinflammation could be crucial in addressing diabetes and its neurological complications. Our results further indicate that exercise interventions could alleviate the T2DM-induced neuroinflammatory state and its associated cognitive deficits by reversing the M1 pro-inflammatory phenotype of hippocampal microglial cells towards the anti-inflammatory M2 phenotype, which, in turn, leads to improved microenvironment of the hippocampal neurogenic niche and neurogenesis.

Furthermore, our findings suggest that exercise upregulates the expression of irisin in the hippocampus of diabetic mice, and blocking irisin signaling can diminish the beneficial effects of exercise on neuroinflammation and AHN. These results indicate that irisin may play a critical role in mediating the interplay between exercise, neuroinflammation, and AHN. Those findings are in agreement with the results of recent studies where irisin plays a pivotal role in managing brain plasticity by inter-organ communications, such as muscle–brain and liver–brain dialogues, during exercise [27,43,62,63]. Irisin, often referred to as the “exercise hormone” [64,65], is upregulated by physical exercise and exhibits beneficial effects in mitigating neuroinflammation, enhancing synaptic plasticity, promoting AHN, and supporting overall cognitive function [27,43,62,63]. Additionally, our results indicate that blocking irisin signaling inhibits the exercise-induced reduction of the TLR4/MyD88/NF-κB pathway. The TLR4/MyD88/NF-κB pathway is recognized as a classical inflammatory signaling cascade that is essential to the regulation of neuroinflammation [35] and neurogenesis [36]. It indicates that this pathway may be engaged in mediating the beneficial effects of exercise on neuroinflammation and neurogenesis in an irisin-dependent manner. This study adds new evidence to the current understanding of how exercise can improve diabetes-associated neuroinflammation and cognitive deficits by promoting the release of irisin and its downstream signaling pathways. These insights suggest that targeting the irisin pathway and its relevant signaling molecules may offer a novel therapeutic approach to mitigating the adverse effects of diabetes on brain function. Given these findings, future research should delve deeper into the molecular mechanisms by which exercise modulates irisin signaling and its subsequent impact on neuroinflammation and neurogenesis, particularly in the context of diabetic conditions. This could pave the way for developing more targeted exercise regimens and therapeutic strategies that leverage the neuroprotective effects of irisin to combat cognitive decline in diabetes.

## 5. Conclusions

In our research, we observed increased neuroinflammation and impaired neurogenesis in the brains of diabetic mice. These changes were accompanied by a significant elevation of TLR4, MyD88, and p-NF-κB proteins. However, exercise training led to improved neuroinflammation and neurogenesis, as well as a reduction in these proteins. Interestingly, when irisin signaling was blocked, the positive effects of exercise on the TLR4/MyD88/NF-κB pathways, as well as neuroinflammation and neurogenesis, were diminished. This suggests that exercise may elevate irisin levels, which, in turn, suppresses TLR4/MyD88/NF-κB signaling, leading to improved neuroinflammation and enhanced hippocampal neurogenesis. Despite this study providing insights into the beneficial effects of exercise, there are certain limitations. Using the cyclo RGDyk peptide to inhibit the irisin receptor αV/β5 could inadvertently affect the expression of another integrin, αV/β3, potentially introducing nonspecific outcomes. Another limitation of our study is that it only included male mice. Given the importance of including both genders in biomedical research, we will incorporate female mice in future studies to provide a comprehensive analysis of gender differences in T2DM development and response to treatments. In order to comprehensively assess the cognitive abilities of T2DM mice, we plan to include additional cognitive tests with minimal physical demands, such as the novel object recognition test, Y-maze, or fear conditioning test, in our future experiments. Additionally, our analysis primarily focused on in vivo studies, lacking a detailed exploration of irisin’s specific actions across diverse hippocampal cell populations, nerve proliferation, and neuronal maturation processes. Future studies should address these gaps, further unraveling the intricate mechanisms underlying irisin’s potential as a therapeutic approach for enhancing neurogenesis in diabetic conditions.

## Figures and Tables

**Table 1:** The sequences of primers used for real-time quantitative reverse transcription polymerase chain reaction.

Name	Primer Sequence (5′ to 3′)
IL-1β	F:TCAGCACCTCACAAGCAGAG R:TTCTTGTGACCCTGAGCGAC
TNF-α	F:TGTCCCTTTCACTCACTGGC R:TCTTCTGCCAGTTCCACGTC

## Data Availability

Code for data cleaning and analysis is provided as part of the replication package. It is available at https://doi.org/10.6084/m9.figshare.25417585.v2 (accessed on 1 August 2024).

## Supplementary Materials

The following supporting information can be downloaded at: https://www.mdpi.com/article/10.3390/biology13100809/s1, Table S1: Comparisons of Western blot and immunofluorescence data between groups in Figure 5. Figure S1: Western blot membrane of DCX (~40 kDa) protein detected with anti-DCX (1:500, sc-271390, Santa Cruz, USA) antibody. Figure S2: Western blot membrane of Iba1 (~16 kDa), IL1β (~17 kDa) and TNF-α (~25 kDa) protein detected with anti- Iba1 (1:1000, AF7143, Beyotime, China), anti-IL1β (1:1000, AF4006, Affinity, China), and anti-TNF-α (1:1000, AF7014, Affinity, China) antibodies. Figure S3: Western blot membrane of FNDC5 (~25kDa), TLR4 (~95 kDa), MyD88 (~40 kDa), p-NF-κB (~65 kDa), and NF-κB (~65 kDa) protein detected with anti-FNDC5 (1:1000, A18107, Abclonal, China), anti-TLR4 (1:500, WL00196, wanleibio, China), anti-MyD88 (1:500, WL02494, wanleibio, China), anti-NF-κB (1:1000, sc-8008, Santa Cruz, USA), and anti- p-NF-κB (1:1000, sc-136548, Santa Cruz, USA) antibody.

## Abbreviations

T2DM	Type 2 diabetic mellitus
STZ	Streptozocin
AHN	Adult hippocampal neurogenesis
TLR4	Toll-like receptor 4
MyD88	Myeloid differential protein-88
NF-κB	Nuclear factor kappa-B
TNF-α	Tumor necrosis factor-α
IL-1β	Interleukin-1β
IFNγ	Interferon-γ
Arg-1	Arginase 1
TGF-β1	Transforming growth factor-β
FBG	Fasting blood glucose
SDS-PAGE	Sodium Dodecyl Sulfate Polyacrylamide Gel Electrophoresis
DAPI	4′,6-diamidino-2-phenylindole
Iba-1	Ionized calcium-binding adapter molecule 1
iNOS	Inducible nitric oxide synthase
CD206	Mannose Receptor
DCX	-doublecortin
FNDC5	Fibronectin type III domain-containing protein 5
ANOVA	Analysis of variance
