# Supplementary material for: Exercise Promotes Hippocampal Neurogenesis in T2DM Mice via Irisin/TLR4/MyD88/NF-κB-Mediated Neuroinflammation Pathway
Source: Biology (Basel). 2024 Oct 10;13(10):809. doi: 10.3390/biology13100809 (PMC11504848; doi:10.3390/biology13100809)
Supplement: Supplementary file 1 [file biology-13-00809-s001.zip › biology-3197233-supplementary.pdf]

Supplementary Materials

Table S1. Comparisons of Western Blot and immunofluorescence data between groups.

		Con	DM	Ex	ExRg
Western Blot					
A	Iba1	1.32±0.70	5.01±0.38*	3.50±0.79$^{*\#}$	5.25±0.52^{*+}
B	DCX+	0.99±0.11	0.16±0.04*	0.87±0.11$^{*\#}$	0.37±0.05^{*+}
Immunofluorescence					
C	Iba1	17.119±0.501	55.023±5.578*	25.085±2.331$^{*\#}$	40.257±4.960$^{*\#+}$
D	DCX+	3.858±0.212	0.684±0.070*	2.605±0.468$^{*\#}$	1.375±0.241$^{*\#+}$
E	Iba1/CD206	1.496±0.156	1.010±0.098*	9.270±0.309$^{*\#}$	3.343±0.668$^{*\#+}$
F	Iba1/iNOS	3.222±0.093	16.805±0.931*	5.896±0.349$^{*\#}$	10.522±0.875$^{*\#+}$

Notes. Data of the overall western blot and Immunofluorescence in Fig 5. (A) Ratio of Iba1 protein among the four groups in Fig 5E. (B) Ratio of DCX protein among the four groups in Fig 5E. (C) Ratio of Iba1 positive area (%) among the four groups. (D) Ratio of DCX positive area (%) among the four groups. (E) Ratio of the relative area of colocalization of Iba1 and CD206 among the four groups. (F) Ratio of the relative area of colocalization of Iba1 and iNOS among the four groups.

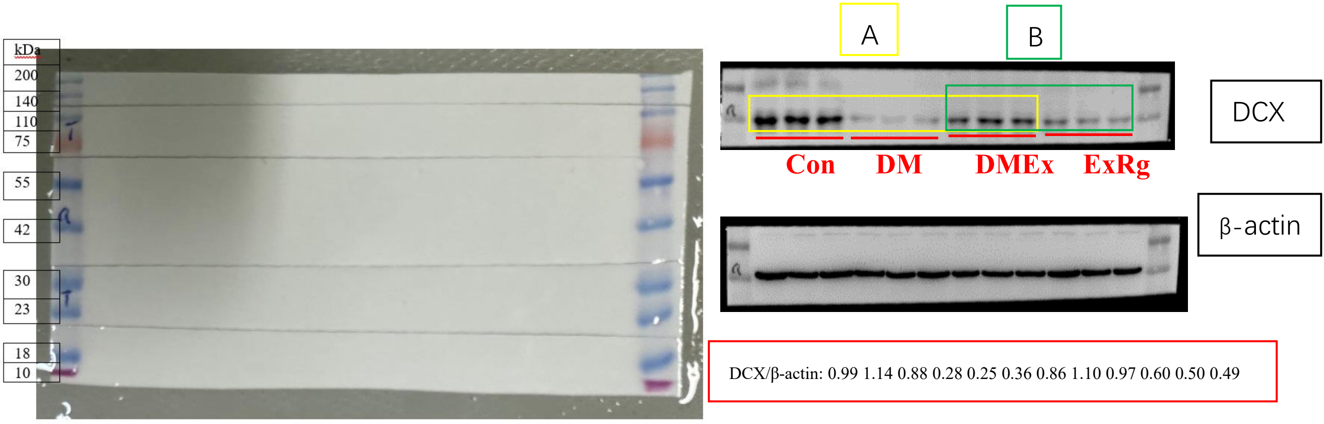

Repeated DCX/β-Actin Ratio			
CON	DM	Ex	ExRg
1.05	0.19	0.81	0.35
0.92	0.19	0.80	0.37
0.96	0.12	0.85	0.40

Figure S1.

(A) DCX protein bands in Figure 1B. (B) DCX protein bands in Figure 5C.

Western blot membrane of DCX (~40 kDa) protein detected with anti-DCX (1:500, sc-271390, Santa Cruz, USA) antibody. Gel-separated proteins were transferred to nitrocellulose membranes by transmembrane (300 mA, 30 min) Membranes, Appropriate secondary antibodies (1:4000, Biodragon) were incubated with the membranes for 1 hour at room temperature. #Weight marker (molecular weight in kDa): Servicebio, 10 to 200 kDa; catalogue number: G2058. Blot images, prior to the densitometry readings, were converted to grayscale with ImageJ (ImageJ v.1.49, National Institutes of Health, Maryland, USA) as follows: Image -> Type –> 8 bit, next: Process -> subtract Background, finally: Use the rectangle box to annotate the strip, Analyze -> Gels -> Select First Lane; Analyze -> Gels -> Plot Lanes.

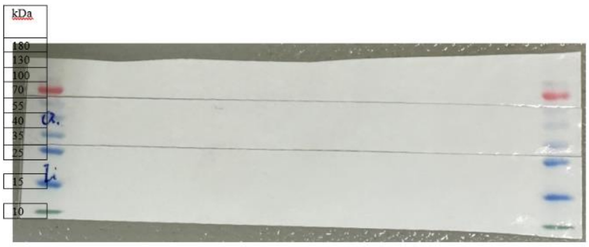

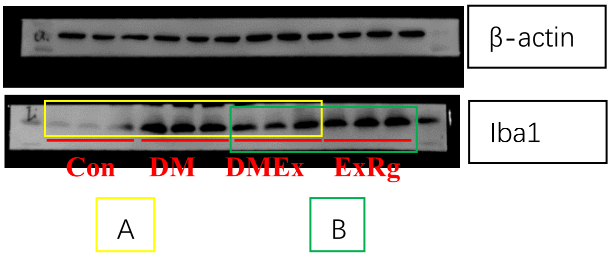

Iba1/β-actin: 0.32 0.53 2.15 4.71 4.73 4.36 2.89 2.70 4.83 5.41 4.69 5.3

Repeated Iba1/β-Actin Ratio			
CON	DM	Ex	ExRg
1.62	5.00	3.15	4.95
1.43	5.15	3.64	5.01
0.94	5.27	4.12	4.72

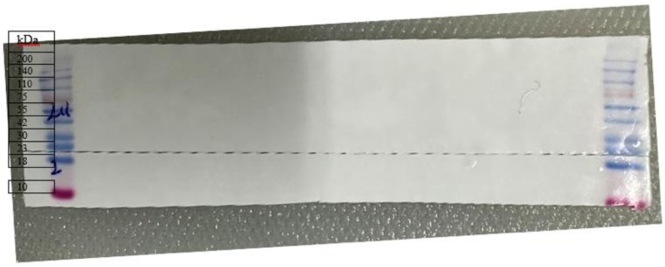

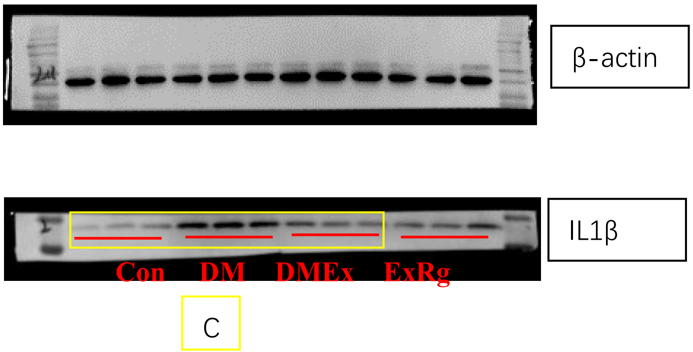

IL1β/β-actin: 0.43 0.96 1.61 4.54 4.18 3.80 2.15 1.89 1.98 2.65 2.91 3.02

Repeated Il1β/β-Actin Ratio		
CON	DM	Ex
1.62	5.00	2.65
1.43	5.15	2.46
0.94	5.27	2.97

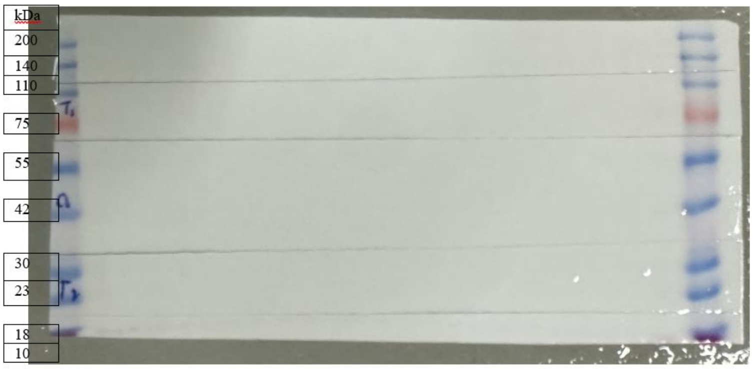

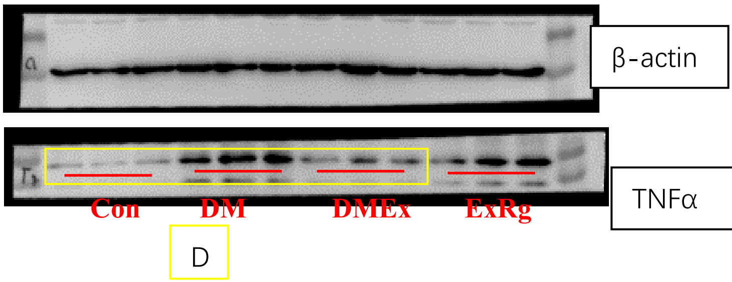

TNFα/β-actin: 1.22 0.70 1.08 3.35 4.01 4.33 2.03 1.70 1.85 2.86 4.16 4.16

Repeated TNFα/β-Actin Ratio		
CON	DM	Ex
1.15	5.14	2.57
0.94	5.28	2.48
1.28	5.95	2.68

Figure S2.

(A) Iba1 protein bands in Figure 2A. (B) Iba1 protein bands in Figure 5C. (C) IL1β protein bands in Figure 2A. (D)TNFα protein bands in Figure 2A.

Western blot membrane of Iba1 (~16 kDa)、IL1β(~17 kDa) and TNF-α(~25 kDa) protein detected with anti- Iba1 (1:1000, AF7143, Beyotime, China) , anti-IL1β(1:1000, AF4006, Affinity, China) and anti-TNF-α (1:1000, AF7014, Affinity, China) antibodies. Gel-separated proteins were transferred to nitrocellulose membranes by transmembrane (300 mA, 30 min) Membranes, Appropriate secondary antibodies (1:4000, Biodragon) were incubated with the membranes for 1 hour at room temperature. #Weight marker (molecular weight in kDa): Vazyme, 10 to 180 kDa; catalogue number: MP102； Servicebio, 10 to 200 kDa; catalogue number: G2058.Blot images, prior to the densitometry readings, were converted to grayscale with ImageJ (ImageJ v.1.49, National Institutes of Health, Maryland, USA) as follows: Image -> Type –> 8 bit, next: Process -> subtract Background, finally: Use the rectangle box to annotate the strip, Analyze -> Gels -> Select First Lane; Analyze -> Gels -> Plot Lanes.

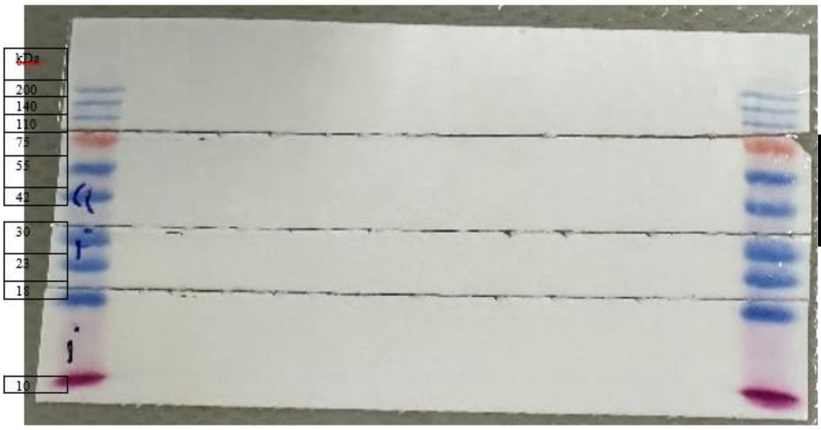

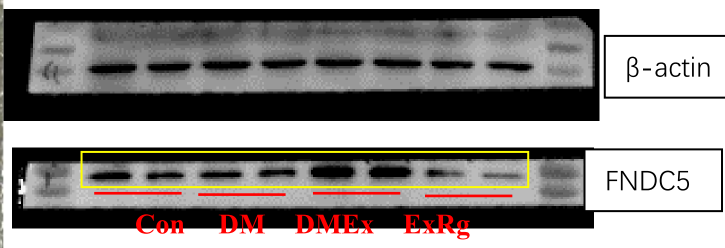

A

FNDC5/βactin: 1.03 0.97 0.95 0.89 1.81 1.57 0.89 0.58

Repeated FNDC5/β-Actin Ratio			
CON	DM	Ex	ExRg
1.02	0.97	1.54	0.40
1.11	1.02	1.54	0.21
1.34	0.91	1.49	0.32
1.23	0.84	1.51	0.38

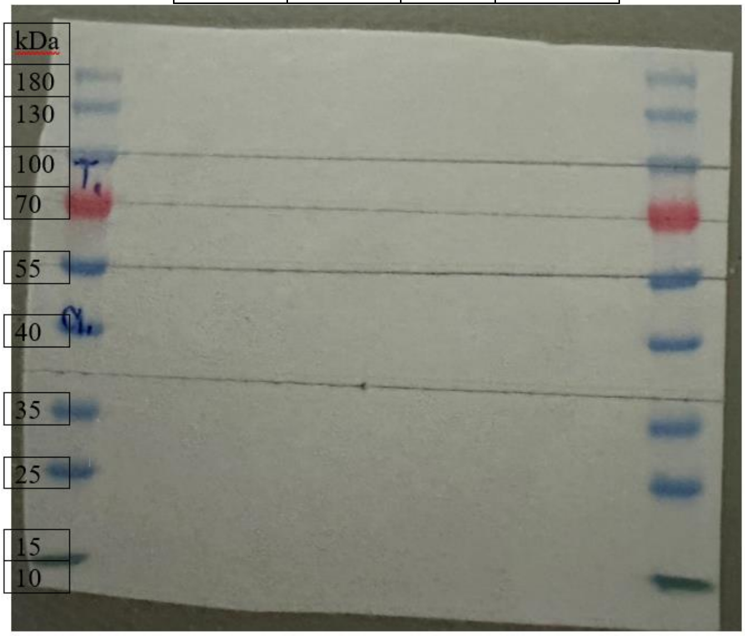

B

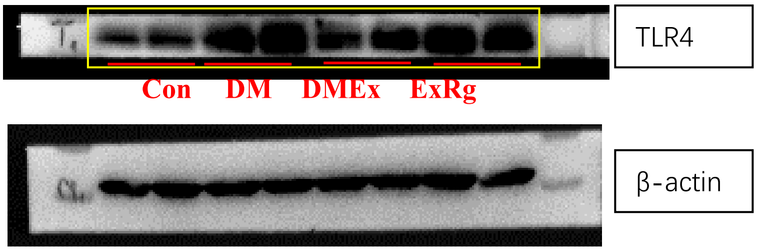

TLR4/β actin: 0.88 1.12 1.76 2.02 1.40 1.47 1.63 1.9

Repeated tlr4/β-Actin Ratio			
CON	DM	Ex	ExRg
0.89	2.36	2.04	2.49
1.41	2.96	2.12	2.67
0.92	2.91	1.96	2.33
1.13	2.65	1.85	2.16

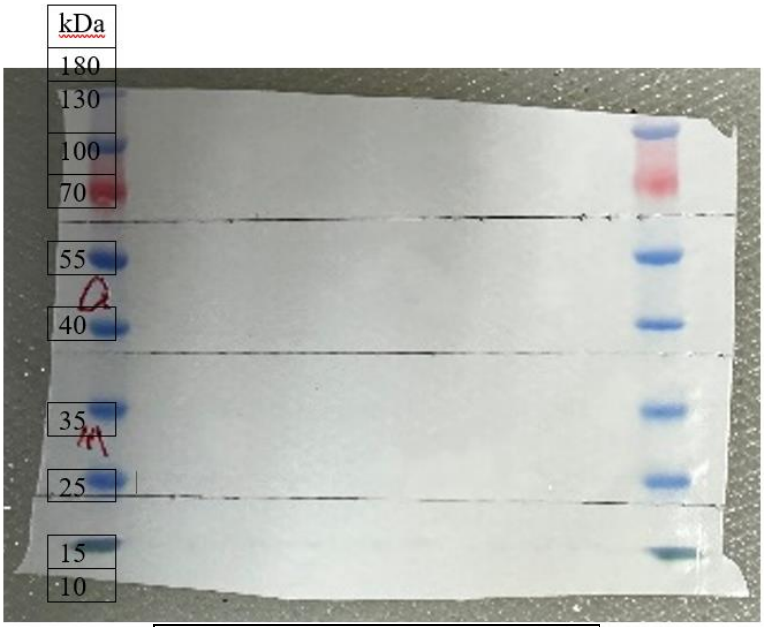

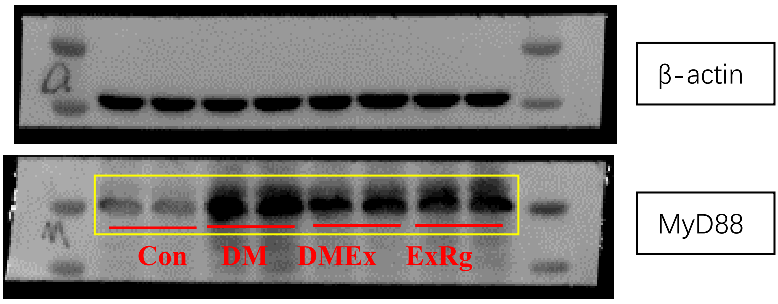

C

TLR4/β actin : 0.97 1.03 3.20 3.41 2.33 2.27 2.59 3.11

Repeated tlr4/β-Actin Ratio			
CON	DM	Ex	ExRg
0.89	2.36	2.04	2.49
1.41	2.96	2.12	2.67
0.92	2.91	1.96	2.33
1.13	2.65	1.85	2.16

D

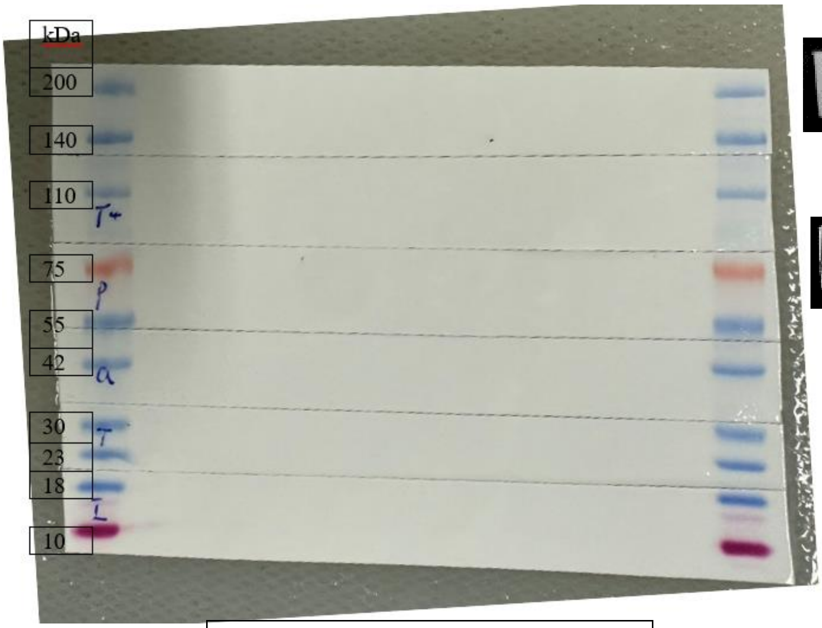

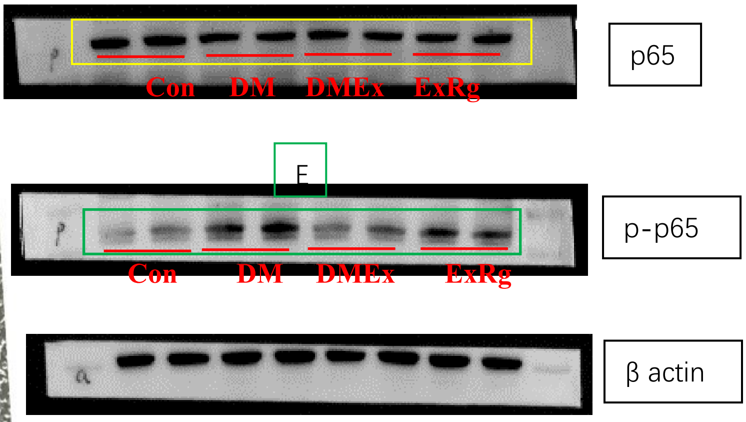

p-p65/p65:0.78 1.22 2.51 2.81 1.51 2.05 2.43 2.11

Repeated p-NF-κB/ NF-κB Ratio			
CON	DM	Ex	ExRg
0.39	3.52	2.39	3.33
0.98	3.34	2.54	3.56
1.06	3.15	2.16	2.73
0.95	3.43	2.06	3.06

Figure S3.

(A) FNDC5 protein bands in Figure 4A. (B) TLR4 protein bands in Figure 4A. (C)MyD88 protein bands in Figure 4A. (D)P65 protein bands in Figure 4A. (E)p-P65 protein bands in Figure 4A. Western blot membrane of FNDC5 (~25kDa), TLR4(~95 kDa) , MyD88(~40 kDa), p-NF-κB (~65 kDa) and NF-κB (~65 kDa) protein detected with anti-FNDC5 (1:1000, A18107, Abclonal, China), anti-TLR4(1:500, WL00196, wanleibio, China) , anti-MyD88(1:500, WL02494, wanleibio, China), anti-NF-κB(1:1000, sc-8008, Santa Cruz, USA) and anti- p-NF-κB (1:1000, sc-136548, Santa Cruz, USA) antibody. Gel-separated proteins were transferred to nitrocellulose membranes by transmembrane (300 mA, 30 min) Membranes, Appropriate secondary antibodies (1:4000, Biodragon) were incubated with the membranes for 1 hour at room temperature. #Weight marker (molecular weight in kDa): Vazyme, 10 to 180 kDa; catalogue number: MP102； Servicebio, 10 to 200 kDa; catalogue number: G2058.Blot images, prior to the densitometry readings, were converted to grayscale with ImageJ (ImageJ v.1.49, National Institutes of Health, Maryland, USA) as follows: Image -> Type –> 8 bit, next: Process -> subtract Background, finally: Use the rectangle box to annotate the strip, Analyze -> Gels -> Select First Lane; Analyze -> Gels -> Plot Lanes.
